# Hypoxia Tolerance of 10 Euphausiid Species in Relation to Vertical Temperature and Oxygen Gradients

**DOI:** 10.3389/fphys.2020.00248

**Published:** 2020-03-24

**Authors:** Nelly Tremblay, Kim Hünerlage, Thorsten Werner

**Affiliations:** ^1^Shelf Sea System Ecology, Alfred-Wegener-Institut Helmholtz-Zentrum für Polar- und Meeresforschung, Helgoland, Germany; ^2^Institute for Sea Fisheries, Thünen Institute, Bremerhaven, Germany; ^3^Nature and Biodiversity Conservation Union, Berlin, Germany

**Keywords:** oxygen minimum zones, diel vertical migration, krill, respiration rate, regulation index

## Abstract

Oxygen Minimum Zones prevail in most of the world’s oceans and are particularly extensive in Eastern Boundary Upwelling Ecosystems such as the Humboldt and the Benguela upwelling systems. In these regions, euphausiids are an important trophic link between primary producers and higher trophic levels. The species are known as pronounced diel vertical migrators, thus facing different levels of oxygen and temperature within a 24 h cycle. Declining oxygen levels may lead to vertically constrained habitats in euphausiids, which consequently will affect several trophic levels in the food web of the respective ecosystem. By using the regulation index (RI), the present study aimed at investigating the hypoxia tolerances of different euphausiid species from Atlantic, Pacific as well as from Polar regions. RI was calculated from 141 data sets and used to differentiate between respiration strategies using median and quartile (Q) values: low degree of oxyregulation (0.25 < RI median < 0.5); high degree of oxyregulation (0.5 < RI median < 1; Q1 > 0.25 or Q3 > 0.75); and metabolic suppression (RI median, Q1 and Q3 < 0). RI values of the polar (*Euphausia superba*, *Thysanoessa inermis*) and sub-tropical (*Euphausia hanseni*, *Nyctiphanes capensis*, and *Nematoscelis megalops*) species indicate a high degree of oxyregulation, whereas almost perfect oxyconformity (RI median ≈ 0; Q1 < 0 and Q3 > 0) was identified for the neritic temperate species *Thysanoessa spinifera* and the tropical species *Euphausia lamelligera.* RI values of *Euphausia distinguenda* and the Humboldt species *Euphausia mucronata* qualified these as metabolic suppressors. RI showed a significant impact of temperature on the respiration strategy of *E. hanseni* from oxyregulation to metabolic suppression. The species’ estimated hypoxia tolerances and the degree of oxyconformity vs. oxyregulation were linked to diel vertical migration behavior and the temperature experienced during migration. The results highlight that the euphausiid species investigated have evolved various strategies to deal with different levels of oxygen, ranging from species showing a high degree of oxyconformity to strong oxyregulation. Neritic species may be more affected by hypoxia, as these are often short-distance-migrators and only adapted to a narrow range of environmental conditions.

## Introduction

Oxygen concentration and water temperature are two important abiotic factors influencing several physiological processes, such as metabolic rate, energy expenditure, as well as the horizontal and vertical distribution of animals living in the world’s oceans (Torres and Childress, 1983; Claireaux and Lagardère, 1999; Ekau et al., 2010). However, both factors are not evenly distributed and temperature and oxygen levels at the surface area are usually higher, compared to deeper water layers. Water temperature is influenced by solar radiation, i.e., latitude and water turbulence. In contrast, oxygen concentration is affected by physical replenishment (mixing), bacterial decomposition and animal respiration. Temperature and oxygen profiles of the water column show a more or less steady decline from upper to deeper water layers (weak thermo- and oxycline) or a more saltatory pattern (strong thermo- and oxycline). In the oceans, the depth and strength of the thermocline vary between season and year. It is semi-permanent in the tropics, variable in temperate regions, and shallow to non-existent in Polar regions. High oxygen concentrations are found at high latitudes, whereas at mid-latitudes, in particular off the western coasts of the continents, oxygen-deficient zones, so-called Oxygen Minimum Zones (OMZs), prevail. Consequently, the ecosystems in the world’s oceans are characterized by distinct oxygen and temperature regimes shaping the different species’ behavior, distribution and physiological processes.

In the anticipated future, anthropogenic induced changes, such as rising nutrient loads coupled with climate change, will cause regional declines in oceanic dissolved oxygen, mainly due to increased stratification and reduced mixing, and an increase in water temperature (Diaz and Rosenberg, 2008; Keeling et al., 2010). Increasing temperature is known to negatively impact the hypoxia tolerance of animals and at the same time raise their energy expenditures. Furthermore, as water temperature rises, oxygen solubility decreases. Thus, decreasing oxygen levels accompanied by increasing temperatures may affect key processes and trophic interactions including community composition, energy flows, migration patterns, and consequently biogeochemical processes (Ekau et al., 2018) and will exert significant pressure on pelagic communities. This applies particularly to planktonic species, such as euphausiids, which cannot, or only to a very limited degree, escape unfavorable environmental conditions (Verheye and Ekau, 2005). As a consequence, it is expected that some areas may experience a shift from an abundant and diverse regime to one that is lean and dominated by vertical migrators (Wishner et al., 2013; Elder and Seibel, 2015).

Evaluation of time series already revealed vertical expansion of OMZs during the last decades (Stramma et al., 2008). It is assumed that these OMZs will further expand, which can happen horizontally into areas previously not experiencing hypoxic conditions, or consist of vertical expansion of an existing OMZ, while coastal hypoxia will increase in extent and severity (Levin, 2018). Compared to other hypoxic habitats, the particular nature of such an OMZ is that it is characterized by moderate to severe hypoxia (<2 mg O_2_ L^–1^) over very large areas (∼8% of total oceanic area; Paulmier and Ruiz-Pino, 2009) and over long time periods. They differ from the “dead zones” phenomena caused by anthropogenic coastal eutrophication found, e.g., in the Gulf of Mexico (Rabalais et al., 2002; Diaz and Rosenberg, 2008). OMZs are permanent midwater features occurring at intermediate depth (300–2,500 m) in most of the oceans (Emelyanov, 2005). The largest and most pronounced OMZs are located in the Northern Indian Ocean, the Eastern Atlantic off northwest Africa, and the Eastern Tropical Pacific (ETP) (Wyrtki, 1962; Kamykowski and Zentara, 1990; Olson et al., 1993). Notably, the OMZ of the ETP and the Eastern Atlantic off northwest Africa have expanded to higher latitudes during the past 50 years (Stramma et al., 2008), suggesting changes in zoogeographic distribution patterns, compression of habitats, and restricted zones of biomass production (Prince and Goodyear, 2006; Koslow et al., 2011; Stramma et al., 2011; Gilly et al., 2013). The shallow and severe OMZ in the ETP is due to the poor lateral ventilation of surface waters (Reid, 1965; Luyten et al., 1983) and the formation of a strong thermocline, which limits O_2_ diffusion into the deeper layers of the ocean (Lavín et al., 2006). Very high temperatures at the surface result in strong stratification, at which the zooplankton aggregate and locally increase the oxygen consumption (Bianchi et al., 2013). At this depth, oxygen is consumed faster than it is replaced by the horizontal mixing of the water mass (Wyrtki, 1962; Fiedler and Talley, 2006; Karstensen et al., 2008), creating the shallow OMZ. The oxygen utilization is particularly enhanced during El Niño-Southern Oscillation and inter-annual changes in upwelling conditions, thus partly explaining the vertical OMZ expansion of the ETP since the 1980s (Ito and Deutsch, 2013).

Compared to Eastern Boundary Upwelling Systems (EBUEs), such as the California, Humboldt, and Benguela Current ecosystems with their pronounced OMZs, the oxygen levels of Polar regions are higher and water temperatures are much lower. No real OMZs exist in these areas and species living there may not be forced to develop adaptations to cope with low oxygen levels. However, mild-hypoxia (50% air saturation) was reported in the Indian sector of the Southern Ocean at depth greater than 500 m (Dehairs et al., 1990) and deoxygenation in the Southern Ocean is currently taking place at 200–400 m depth between 50 and 60° of latitude (Matear et al., 2000; Aoki, 2005). In the Artic, the potential effects of global warming and changes in deep-sea circulation on the oxygenation of the deep ocean is monitored continuously in Fram Strait, West Spitsbergen, the only deep connection between the central Arctic Ocean and the Nordic Seas (Friedrich et al., 2014). The Arctic ecosystem is far from being classified as hypoxic, but strong increase in the annual mean net heat transport within the waters of the West Spitsbergen Current could potentially affect oxygen levels to less than 80% air saturation.

Euphausiids, or krill, are distributed ubiquitously across the globe and often dominate zooplankton communities in terms of abundance and biomass throughout the world‘s oceans. Euphausiids form a pivotal component of many food webs and are known as pronounced diel vertical migrators, thereby contributing to the vertical flux of carbon and facing different levels of oxygen and temperature within a 12 h period. During diel vertical migration (DVM), many euphausiid species cross pronounced gradients of temperature, salinity, and oxygen indicating that these species must be of a broad ecophysiological plasticity. In this regard, euphausiids are ideal model organisms for studying the interactions between organismal and environmental variability (Mangel and Nicol, 2000). Euphausiids and other taxa living in areas with pronounced OMZs have to physiologically and/or behaviorally adapt to low oxygen levels or will be excluded from these areas or at least their vertical distribution ranges will be limited. A typical euphausiid DVM pattern consists of an upward migration at dusk to feed in the upper, productive layers of the oceans, and a downward movement at dawn to avoid visual predators (Zaret and Suffern, 1976; Ohman, 1984), decreasing at the same time their metabolic rates due to the lower water temperature and O_2_ concentrations (McLaren, 1963; Enright, 1977). Euphausiids channel energy from lower (phytoplankton, small zooplankton) to higher (fish, birds, and even whales) trophic levels. Accordingly, as varying oxygen and temperature levels will likely alter these species’ vertical and horizontal distribution ranges, this may impact a larger part of the whole food web, and even impinge on fisheries yield.

Adaptations of animals to low dissolved oxygen concentrations are driven by strong selective pressures to maintain aerobic metabolism (Seibel, 2011). Most animals facing low oxygen concentrations respond either by decreasing their oxygen consumption rates, known as oxyconformity, or by maintaining a constant oxygen uptake irrespective of the ambient oxygen levels, known as oxyregulation. However, as analyzed mathematically by Cobbs and Alexander (2018) using/applying seven different functions and as discussed by Wood (2018), animals seldom show perfect oxyconformity or oxyregulation. Accordingly, species’ metabolic responses to declining oxygen levels lay somewhere between the two ends of this continuum (Mueller and Seymour, 2011). Furthermore, at a certain species-specific oxygen pressure, animals are unable to maintain their normoxic metabolic rate and have to goose anaerobic metabolism. This point is called ‘critical oxygen partial pressure’ (*P*_*crit*_) and can be determined by analyzing the response of the metabolic rate (respiration) to declining oxygen concentrations. Oxyconformers do not regulate their oxygen demand as, physiologically, these species do not need to enhance the transport of oxygen to the metabolizing tissues when oxygen is decreasing. Thus, the capability of an animal to either regulate its oxygen uptake in combination with the *P*_*crit*_ value or reduce its respiration rate when ambient oxygen levels decrease provides meaningful information about their ability to survive hypoxic events and represent an important ecological tipping point to understand the resilience of populations to declining levels of oxygen (Mueller and Seymour, 2011). A third strategy called metabolic suppression entails the suppression of total energy consumption by shutting down intensive energy demanding processes (Seibel, 2011; Seibel et al., 2016). This strategy has been observed in euphausiid species inhabiting regions where oxygen decline was faster than euphausiid oxygen demands (Seibel et al., 2016).

In this paper, we aim to characterize the hypoxia tolerance of 10 dominant euphausiid species from the Atlantic and the Pacific Ocean, including three prominent EBUEs (Benguela, California, and the Humboldt Current system), and both Polar regions at *in situ* temperatures by analyzing the regulation index (RI) to explain the DVM behavior in their habitat.

## Materials and Methods

**FIGURE 1:**
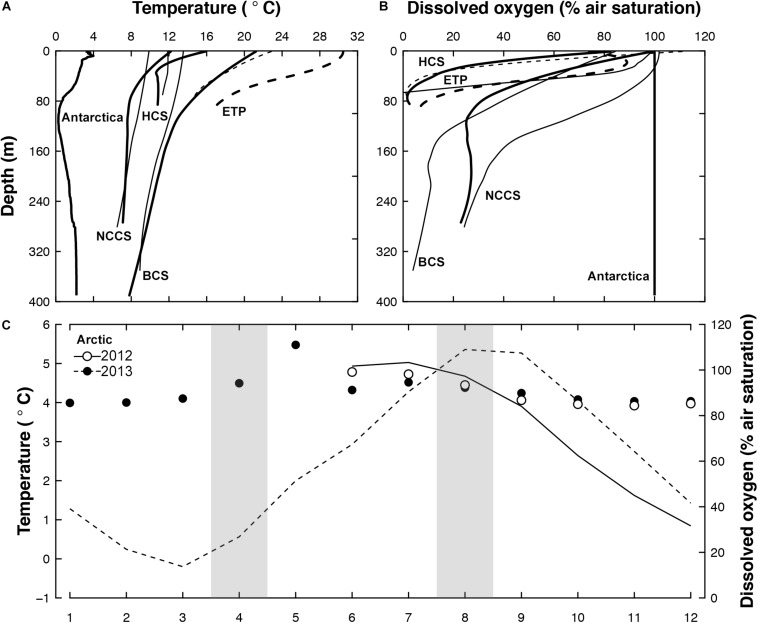
Temperature and oxygen during sampling periods. Depth profiles of **(A)** mean water temperature and **(B)** dissolved oxygen in Antarctica, the Humboldt current system [HCS], the Eastern Tropical Pacific [ETP], the Benguela Current System [BCS], and the Northern California Current System [NCCS]. Bold lines represent summer conditions and dashed lines the ETP area. **(C)** Monthly mean temperature (line) and dissolved oxygen (dots) in the Arctic with sampling periods shaded in gray. Data were compiled from the AWIPEV underwater observatory located at 12 m water depth (Fischer et al., 2018a, b).

Ten euphausiid species were collected between 2010 and 2013 during several small- or large-scale expeditions to the Benguela, California, and Humboldt Current systems (BCS, CCS, and HCS), the Eastern Tropical Pacific (ETP), the Arctic and the Antarctic (details compiled in Table 1). Polar (Antarctic and Arctic: between −0.5°C and 5.5°C), temperate (NCCS and HCS: between 6.5°C and 15.1°C), sub-tropical (BCS: between 8.0°C and 21°C), and tropical (ETP: between 14.6°C and 30.2°C) temperature gradients as well as different hypoxic conditions (severe and shallow: ETP and HCS; severe and deep: BCS; moderate: NCCS; and non-existent: Antarctica and Arctic) are thus covered by the habitat of the species studied (Figure 1).

**TABLE 1:** From North to South: Sampling areas (latitude/longitude), species names, number of individuals analyzed (*n*), specimens mean weight (±standard deviation; W, wet weight; D, dry weight) and respiration measurement information in the Benguela, Northern California, and Humboldt Current systems (BCS, CCS, and HCS), in the Eastern Tropical Pacific (ETP), in the Arctic (Kongsfjord, Spitsbergen) and in Antarctica (South Georgia).

Area	Date	Latitude/longitude	Cruise/boat	Sampling gear	Species	Measurement			
						*n*	Weight (mg)	Temperature (°C)	Mean duration (h)
Arctic	Aug 2012	78.95° N/12.33°E	MS Teisten	1-m^2^ Tucker trawl,	*Thysanoessa inermis*	6	90 ± 33(W)	2	27.8
	Apr 2013			1,000 μm mesh size, soft		4	84 ± 2(W)	6	9.2
	Aug 2013			cod-end, speed of two knots		8	66 ± 14(W)	8	6.0
						4	124 ± 25(W)	10	4.3
NCCS	Sep 2011	44.7°N/124.7°W	RV Elahka	Bongo, 0.6 m diameter,	*Euphausia pacifica*	17	9 ± 6(D)	10	3.8
	Apr 2012			333 μm black mesh, non-filtering cod-end, obliquely to ∼25 m	*Thysanoessa spinifera*	6	37 ± 21(D)	10	3.5
ETP	Feb 2012	19.2°N/104.7°W	Fiberglass boat (6 m)	1 m diameter, 3 m long, 300 μm mesh, non-filtering cod-end, obliquely	*Euphausia distinguenda*	10	3.0 ± 0.5(D)	20	3.6
				∼30 m,speed of 4 km h^–1^, 10 min	*Euphausia lamelligera*	3	0.7 ± 0.3(D)	20	3.2
BCS	Sep 2010	23°S/13°W	RSS Discovery	1-m^2^ Multiple Opening and Closing	*Euphausia hanseni*	4	122 ± 21(W)	5	3.6
	Feb 2011		RV Maria	Net and Environmental Sensor		22	96 ± 25(W)	10	4.3
			S. Merian	System, 2,000 μm mesh size, soft		4	115 ± 11(W)	15	3.3
	Sep 2013		RV Meteor	cloth cod-end, speed of two knots	*Nematoscelis megalops*	11	53 ± 9(W)	20	3.3
						2	110 ± 13(W)	10	0.7
					*Nyctiphanes capensis*	13	31 ± 8(W)	10	13.1
HCS	Aug 2011	36.5°S/73.1°W	RV Kay-Kay II	1 m diameter, 5 m long, 300 μm black mesh, non-filtering cod end	*Euphausia mucronata*	5	6 ± 4(D)	8	11.8
Antarctica	Jan 2012	53–55°S/37–41°W	RRS James Clark Ross	Rectangular midwater trawl of 8 m^2^ mouth area	*Euphausia superba*	21	296 ± 64(D)	4	12.7
									

All samplings were executed during night time, when euphausiids are more abundant at the surface, to avoid overstressing the experimental animals by reducing catch time. Live adult euphausiids, in healthy condition (showing a lot of movement and with no visible damage), were manually sorted into bins filled with filtered seawater at *in situ* temperature and acclimated for at least 6 (CCS, HCS, ETP, and Antarctica) or 12 h (BCS and Arctic) prior to starting respirometry procedures to make sure that all animals are in a post-absorptive state.

### Respirometry

The measurements were conducted in the dark to mimic the conditions of the time of the day when euphausiids should be in deeper water and hypoxic conditions when hypoxia applied to the area. The same closed configuration system, chamber volume (20 mL; except for Antarctica where chamber volume was 250 mL to account for the larger size of *Euphausia superba*) and measurement method were used in all areas. The oxygen level within the chamber decreased as the effect of respiration. Measurements were carried out at *in situ* temperature for the 10 species, and at four different temperatures for *Thysanoessa inermis* (Arctic; 2, 4, 8, and 10°C) and *Euphausia hanseni* (BCS; 5, 10, 15, and 20°C) to assess how temperature modulates intraspecific hypoxia tolerance (Table 1). Both species were acclimated at a rate of 1°C h^–1^ to colder and warmer temperatures for at least 12 h after completion of the 12 h post-capture acclimation. The thermal ramp steepness and amplitude took into consideration the vertical migration temperature gradient that *E. hanseni* experience during DVM (Werner and Buchholz, 2013) and the Arrhenius breakpoint temperature (12°C) of *T. inermis* (Huenerlage and Buchholz, 2015; Huenerlage et al., 2016).

OXY-4 or -10 channel PreSens Oxygen Measurement system (Regensburg, Germany) was used with dipping probes DP-PSt3 or planar oxygen-sensitive foils PSt3 integrated in the chambers. Probes and foils were calibrated at *in situ* temperature prior to measurements at 0% air saturation with sodium sulfite (Na_2_SO_3_; 1 g in 100 mL water) and at 100% air saturation with air-saturated water (10 min after air injection in stirred water for 20 min). The system was equipped with four (OXY-4) or ten (OXY-10) chambers including respectively one or two blanks (for seawater bacterial oxygen demand). All chambers were filled with filtered local seawater at 100% air saturation and the oxygen concentration in each chamber was measured every 15 or 30 s. The first 30 min of each measurement were discarded to allow acclimation to chamber. Movements of the pleopods and/or heartbeats of the animals were visually monitored to make sure that they were alive during the entire duration of the measurement. Wet or dry (48 h at 50°C) weight of the preserved animal was measured after completion of the respiration measurement (information provided in Table 1). All respiration rates were reported as mL O_2_ h^–1^g wet weight^–1^. For some species, only the dry weight was available and it was converted to wet weight using the euphausiids conversion equation of Kiørboe (2013) to allow comparison among the 10 species. The programming environment for data analyses and graphics R (R Core Team^1^) was used to calculate the RI [see section “Regulation Index (RI)”] [script provided as Supplementary Material (see Supplementary Data Sheet 1)].

### Regulation Index (RI)

**FIGURE 2:**
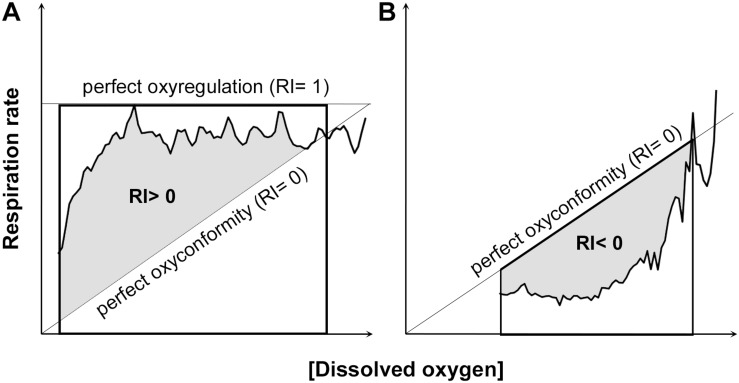
Examples of regulation index (RI) calculations. As shown by Mueller and Seymour (2011), the area under the curve and above the linear regression of perfect oxyconformity was used to calculate a positive RI **(A)**. In contrast, a negative RI was calculated as the area bounded by the area above the curve and the linear regression of perfect oxyconformity **(B)**.

Mueller and Seymour (2011) were the first to propose the use of the RI to assess regulation ability of aquatic organisms that do not present a clear critical oxygen partial pressure (P_crit_) in their respiration pattern. The authors advised to fit a curve (straight line, quadratic or one-phase association) with the highest *r*^2^ to the respiration rate data for each individual plotted against the whole oxygen concentration range measured within the respiration chamber (ideally from 100 to 0% air saturation). RI corresponded to the proportion of the area bounded by a linear regression that represented how respiration rates would decline if the animals showed complete oxyconformity (perfect oxyconformity; RI = 0) and a horizontal line at maximum oxygen consumption (perfect regulation; RI = 1). The perfect oxyconformity linear regression assumes zero respiration rate at 0% air saturation (Figure 2A).

The present work used 141 respiration data sets [available in Supplementary Material (see Supplementary Data Sheet 1)], in which the experimental oxygen concentration dropped to ≤50% of the respective experiments’ start concentration. In order to reduce user interpretation in calculating RI by mean of the best fitted curve, no parametric model was fitted to the original data sets. The area under curve was computed using R package “MESS” (Ekstrøm, 2018) with the natural spline interpolation (loess) for dissolved oxygen concentration as *x*-values and respiration rates as *y*-values. The same procedure was conducted with the linear regression that represents perfect oxyconformity and perfect oxyregulation. When the natural spline interpolation of the data was below the linear regression of perfect oxyconformity, RI became negative and was calculated from the area bounded by the horizontal line at *y* = 0 and the linear regression that represented perfect oxyconformity (Figure 2B). A negative RI value can thus be interpreted as hypoxia-sensitivity (Alexander and McMahon, 2004), but could be an indication of metabolic suppression, as respiration rates are significantly reduced. Respiration strategies using median and quartile values were defined as: low degree of oxyregulation (0.25 < RI median < 0.5); high degree of oxyregulation (0.5 < RI median < 1; Q1 > 0.25 or Q3 > 0.75); oxyconformity (RI median≈0; Q1 < 0 and Q3 > 0) and metabolic suppression (RI median, Q1 and Q3 < 0).

### Statistical Analysis

All statistics and figures were done with R (R Core Team, 2020). For interspecific (*in situ* temperature) and intraspecific (among temperature for *E. hanseni* and *T. inermis*) hypoxia tolerance comparison, the non-parametric Kruskal–Wallis test was conducted (normality and variance homogeneity were not met). Significant level of all comparisons was fixed at 95% (*p* < 0.05). For *post hoc* comparison a multiple comparison test from the package “pgirmess” (Giraudoux, 2018) was applied.

## Results

**FIGURE 3:**
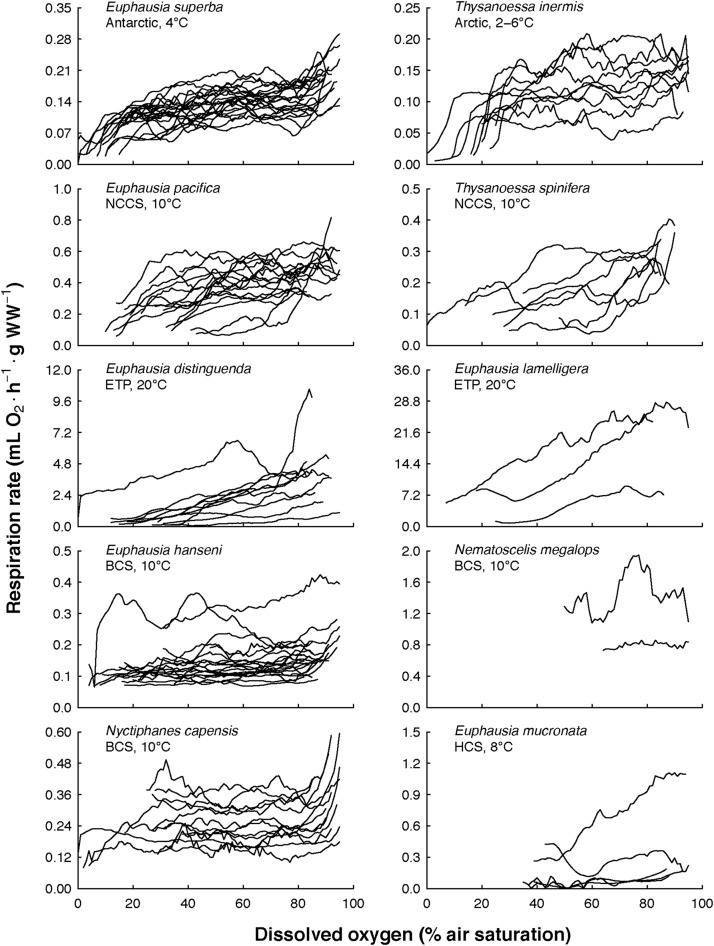
Euphausiids’ respiration rates over decreasing dissolved oxygen concentration at *in situ* temperature. The 10 euphausiids species were from both Polar regions, three major Eastern Boundary Upwelling Systems (NCCS, Northern California Current System; BCS, Benguela Current System; HCS, Humboldt Current System), and one tropical region (ETP, Eastern Tropical Pacific).

**FIGURE 4:**
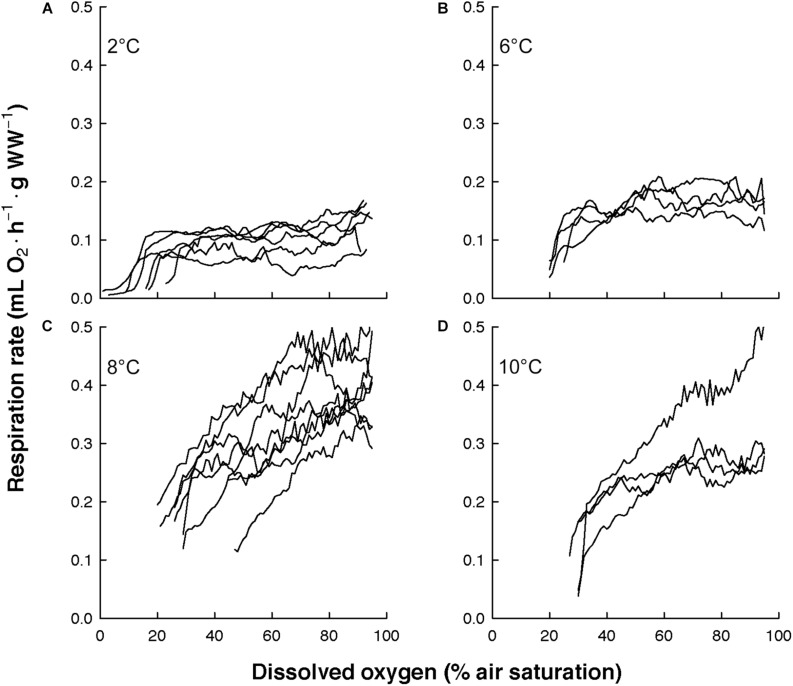
Respiration rates of *Thysanoessa inermis* over decreasing dissolved oxygen concentration at four temperatures. **(A)** at 2°C (*n* = 6), **(B)** at 6°C (*n* = 4), **(C)** at 8°C (*n* = 8), and **(D)** at 10°C (*n* = 4).

**FIGURE 5:**
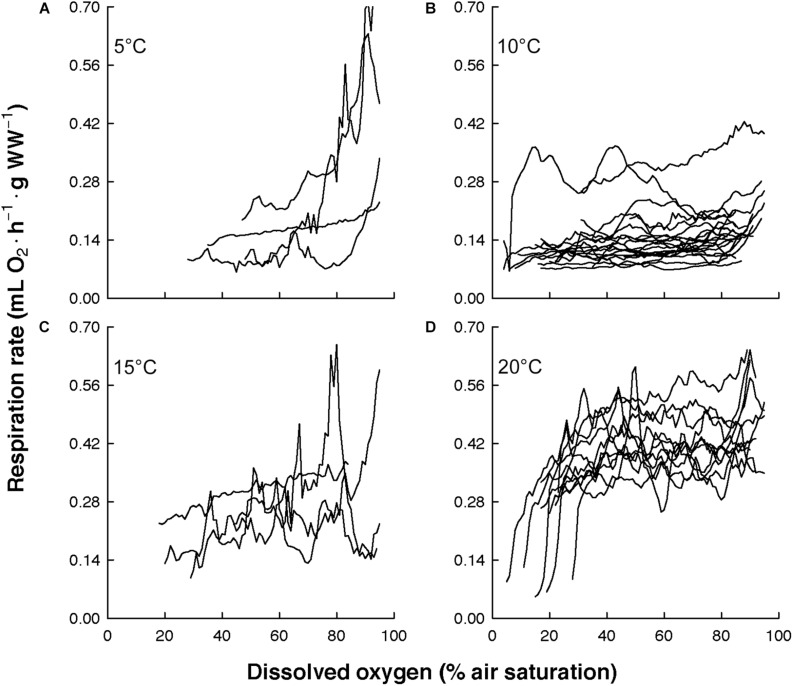
Respiration rates of *Euphausia hanseni* over decreasing dissolved oxygen concentration at four temperatures. **(A)** at 5°C (*n* = 4), **(B)** at 10°C (*n* = 22), **(C)** at 15°C (*n* = 4), and **(D)** at 20°C (*n* = 11).

The overall view of the euphausiids’ respiration rates over decreasing dissolved oxygen concentration at *in situ* temperature shows different magnitude and patterns (Figure 3). Comparing this magnitude by area, the highest respiration rates were observed in *Euphausia pacifica* (NCCS), *Euphausia lamelligera* (ETP), and *Nematoscelis megalops* (BCS). Different magnitude and patterns were also seen intraspecifically when *T. inermis* and *E. hanseni* were acclimated at lower or higher temperatures (Figures 4, 5). The respiration rates of *T. inermis* increased at 8 and 10°C (Figures 4C,D) in comparison to 2 and 6°C (Figures 4A,B) during the whole oxygen range measured. For *E. hanseni*, respiration rates were similar at all temperatures in the high-oxygen levels between 80 and 100% air saturation (Figure 5).

**FIGURE 6:**
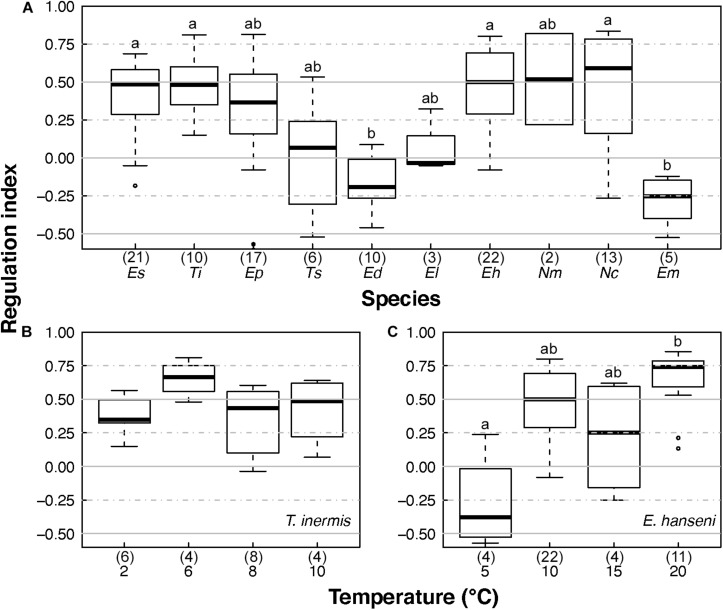
Euphausiids’ regulation indices at *in situ* temperature and after temperature acclimation. **(A)** Regulation indices of the 10 euphausiid species investigated at *in situ* temperature, **(B)** regulation indices of *T. inermis* acclimated at 2, 6, 8, and 10°C, and **(C)** regulation indices of *E. hanseni* acclimated at 5, 10, 15, and 20°C. *Es*, *E. superba*; *Ti*, *T. inermis*; *Ep*, *E. pacifica*; *Ts*, *T. spinifera*; *Ed*, *E. distinguenda*; *El*, *E. lamelligera*; *Eh*, *E. hanseni*; *Nm*, *N. megalops*; *Nc*, *N. capensis*; *Em*, *E. mucronata*. Numbers in parentheses give the numbers of samples analyzed. Horizontal bars in the box plots indicate the median. The upper and lower edges of the rectangles show the first and third quartiles, respectively. Vertical error bars extend to the lowest and highest values in a 1.5-fold inter-quartile range (R Core Team, 2020). Lower case letters indicate significant differences.

The RI of *Euphausia superba* (Antarctica), *Thysanoessa inermis* (Arctic), *Euphausia hanseni* (BCS), and *Nyctiphanes capensis* (BCS) were significantly higher than the RI of the tropical and temperate species *Euphausia distinguenda* (ETP) and *Euphausia mucronata* (HCS), respectively (Figure 6A, χ*^2^* = 56.05, *p* < 0.000, Table 2). Median RI values ≥0.5 of the polar (*E. superba*, *T. inermis*), temperate (*E. pacifica*), and sub-tropical (*E. hanseni*, *N. megalops*, and *N. capensis*) species indicated a high degree of oxyregulation, whereas the neritic temperate (*T. spinifera*) and tropical (*E. lamelligera*) species showed a low regulation ability as RI values fluctuated between −0.25 and 0.25 (Figure 6A and Table 2). Quartiles values below and above 0 of *T. spinifer*a and *E. lamelligera* indicate almost perfect oxyconformity of these species. The oceanic tropical species *E. distinguenda* and the Humboldt endemic species *E. mucronata* were qualified as metabolic suppressors with RI median and quartile values well below 0 (Figure 6A and Table 2).

**TABLE 2:** Regulation index (RI) for 10 euphausiid species investigated.

	Temperature	RI			
Species	(°C)	Median	Quartile 1	Quartile 3	Strategy
*Euphausia superba*	4	0.48	0.29	0.58	High oxyregulation
*Thysanoessa inermis*	2	0.34	0.32	0.46	Low oxyregulation
	2–6	0.48	0.35	0.60	High oxyregulation
	6	0.66	0.60	0.72	High oxyregulation
	8	0.43	0.17	0.54	Low oxyregulation
	10	0.49	0.30	0.60	High oxyregulation
*Euphausia pacifica*	10	0.36	0.16	0.55	Low oxyregulation
*Thysanoessa spinifera*	10	0.07	–0.31	0.24	Conformity
*Euphausia distinguenda*	20	–0.19	–0.26	–0.03	Metabolic suppression
*Euphausia lamelligera*	20	–0.03	–0.04	0.14	Conformity
*Euphausia hanseni*	5	–0.38	–0.50	–0.15	Metabolic suppression
	10	0.50	0.29	0.69	High oxyregulation
	15	0.25	–0.11	0.58	Conformity/regulation
	20	0.74	0.59	0.79	High oxyregulation
*Nematoscelis megalops*	10	0.52	0.37	0.67	High oxyregulation
*Nyctiphanes capensis*	10	0.59	0.16	0.78	High oxyregulation
*Euphausia mucronata*	8	–0.26	–0.40	–0.15	Metabolic suppression

*RI was calculated from 141 data sets and used to differentiate between respiration strategies using median and quartile values: low degree of oxyregulation (0.25 < RI median < 0.5); high degree of oxyregulation (0.5 < RI median < 1; Q1 > 0.25 or Q3 > 0.75); oxyconformity (RI median≈0; Q1 < 0 and Q3 > 0), and metabolic suppression (RI median, Q1 and Q3 < 0).*

Regulation index did not change significantly with temperature for *T. inermis* (Figure 6B), but it did in *E. hanseni* from high oxyregulation to metabolic suppression, when acclimation temperature was decreased to 5°C (compared to 20°C; χ^2^ = 14.53, *p* = 0.002; Figure 6C).

## Discussion

Euphausiids and other zooplankton taxa perform DVM to feed on the phytoplankton-rich upper water layers during night time and to reduce mortality from visual predation during the day. These benefits are counteracted by higher energy demands due to increased swimming speeds and higher water temperatures in upper water layers and reduced growth and reproduction rates in deeper, cold water layers. Thus, animals performing DVM have to compensate with increased energy expenditures. Furthermore, they must have evolved physiological and behavioral adaptations to the strong gradients of oxygen and temperature in the water column. As some species suppress their metabolism (Seibel et al., 2016), determination of metabolic rates of diel vertical migrators is crucial to assess the role and quantify the contribution of these animals to the downward transport of carbon and thus carbon fluxes in the oceans. The environmental conditions prevailing in the different ecosystems in terms of oxygen availability and vertical temperature profiles seem to have caused specific physiological adaptations in euphausiids – mostly irrespective of the actual oxygen and temperature level and the time spent in the OMZs. Species which come across the shallowest severe hypoxia levels during their DVM show metabolic suppression [in the Humboldt Current System (HCS) and ETP]. In contrast, the three species from the Benguela Current System (BCS), characterized by a deeper OMZ, maintain constant oxygen uptakes irrespective of the ambient oxygen levels. These differences may indicate that a steep decline in oxygen levels constitutes a physiological threshold at which euphausiids must significantly shut down their metabolic functions (Seibel et al., 2016).

### Shallow OMZs

The hypoxia tolerance of the euphausiid species adapted to the OMZs of the HCS and the ETP was assessed using the RI. In the literature, typical low *P*_*crit*_ between 0.6 and 1.7 kPa at 13°C and 23°C (corresponding to 3% and 8% air saturation) were obtained by Teal and Carey (1967) and Kiko et al. (2016), who were working on *Euphausia mucronata* from the HCS. This euphausiid species performs extensive DVM down to 250 m into the OMZ in all seasons (Escribano et al., 2000; Antezana,2002b). However, highest abundances of this species occur in areas where the upper boundary of the OMZ is deeper (Escribano et al., 2000). During the warm season at 12°C and 13°C, *E. mucronata* maintains the same rate regardless of whether exposed to surface *p*O_2_ (70% air saturation or 17 kPa), or to *p*O_2_ typical for OMZ layers (20% air saturation or 4 kPa; Antezana,2002a; Donoso and Escribano, 2014). The temperature used by the authors cited above represents the warmer range in subsurface water of the area (50–200 m), while the present study was simulating the coldest temperature that can be encountered in the same water depth or at surface during “normal” or cold (La Niña) years between 40°S and 17°S off Chile (Strub et al., 1998). As seen from the changes in RI following the decreasing temperature in *Euphausia hanseni* from the BCS, the OMZ-adapted species of the genus *Euphausia* may regulate their metabolic rates when exposed to warmer surface temperature and tend to conform or suppress their metabolism when exposed to the colder thermal limit of their deeper habitat. This may explain why no oxyregulation pattern at all was observed at 8°C in *E. mucronata*, despite the long duration of the measurement.

The tendency to conform or suppress the metabolism at colder temperature may be also true for *Euphausia distinguenda* from the ETP (corresponding to sub-surface temperature) as shown here with our measurement at 20°C. From field samples collected at different depths above and into the OMZ of the ETP off Mexico, Herrera et al. (2019) observed the highest specific Electron Transfer System (ETS) activity between 3.20 and 3.93 mL O_2_ L^–1^ (48 to 58% air saturation) at 25°C, meaning that the species was still relying on aerobic processes half-way within the oxycline. This ETP species is also reported in the OMZ of the HCS (Antezana, 2009). Both *E. distinguenda* and *E. mucronata* possess larger gills relative to their body size (Antezana,2002a), increasing contact surface for O_2_ diffusion from the hypoxic environment. Antezana (2009) also observed that both were among the last OMZ species to begin their ascent to the surface at dusk in the HCS, thus extending the deep hypoxic residence time to a maximum. Habitat segregation was suggested to explain this behavior, which consists in avoiding spatial and temporal co-occurrence with other species within the same area. This finding was based on body and gills size analysis, feeding appendages, and HCS food resources. *Euphausia lamelligera*, the other ETP species also endemic to the OMZ, dominates the neritic zone while *E. distinguenda* distributes more in oceanic waters (Brinton, 1962, 1979; Färber-Lorda et al., 1994, 2004, 2010). Because of its neritic preference, *E. lamelligera* does not migrate as much as *E. distinguenda*, explaining probably why this species is almost a perfect oxyconformer rather than a metabolic suppressor. Both species are thus highly hypoxia tolerant, reflected mainly by their low RI. As high temperature pushes physiological limits, a small sub-mesoscale oxygen variability in the ETP of ≤1% could affect their vertical and horizontal distribution (Wishner et al., 2018). Accordingly, even small changes in oxygen availability may exert strong pressure on these animals, leading to unexpected changes in ecosystem structure and functioning in the near future. Species of the ETP are adapted to low oxygen and high temperature, but as they live at the edge of their maximum thermal limit, further warming could have negative impact on the fitness of both species as their higher brood size depends on the coastal upwelling dynamics between January and June (Ambriz-Arreola et al., 2015, 2018). A negative RI was initially not proposed by Mueller and Seymour (2011) when developing the RI as a new method to assess hypoxia tolerance of aquatic ectotherms. However, as shown for weak oxyregulating (or perfect oxyconformers) and metabolic suppressing species presented in this study, a negative RI is relevant and thus presents a further development for the use of this index. The corroboration of the presence of metabolism suppression associated with a negative RI remains to be shown looking at physiological metabolic markers (e.g., enzymatic activity, ATP production, anaerobic end-products). This pattern was previously described as hypoxia-sensitivity by Alexander and McMahon (2004).

### Deep OMZ

In the BCS, *E. hanseni* and *Nematoscelis megalops* dominate the shelf break surroundings, i.e., partly sharing one habitat in this upwelling region (Barange and Stuart, 1991; Barange et al., 1991). The species *E. hanseni* performs extensive DVM from 0 to 200 and even 1,000 m water depth (Barange, 1990; Barange and Stuart, 1991; Barange and Pillar, 1992; Werner and Buchholz, 2013), while *N. megalops* is characterized as a weak migrator (Werner and Buchholz, 2013). In contrast to *E. hanseni*, *N. megalops* has a broader distribution and can be found at both sides of the equator: in the mid-latitude zones of the subtropical-temperate North Atlantic (10–60°N), in the warm-temperate belts of the South Atlantic, the Indian Ocean and the South Pacific (35–50°S), in the Mediterranean Sea (e.g., Gopalakrishnan, 1974), in subarctic regions (Zhukova et al., 2009), even up to 79°N in the high Arctic Kongsfjord (Buchholz et al., 2009; Huenerlage and Buchholz, 2015). Morphologically and ecologically, *N. megalops* is very similar to *Nematoscelis difficilis* from the ETP and California Current System (Karedin, 1971; Gopalakrishnan, 1974, 1975). Those species are observed within the OMZ, but in its upper boundary (Tremblay et al., 2010; Werner and Buchholz, 2013), probably taking advantage of the accumulation of organisms to actively feed. The third species *Nyctiphanes capensis* shows extraordinarily high abundances over the Namibian shelf in water <200 m depth (Barange and Stuart, 1991; Barange and Pillar, 1992).

*E. hanseni* and *N. capensis* have one of the highest RI values of all species analyzed meaning that they cover their energy requirements at low oxygen levels in the coldest temperature experienced in their habitat. RI values were enhanced in *E. hanseni* acclimated at 20°C compared to 10°C, which is similar to results of Teal and Carey (1967) and Kiko et al. (2016) at sub-surface temperature conditions with the species *E. mucronata* from the HCS. However, the respiration rate of *N. megalops* investigated here was 10-fold higher than of *E. hanseni* and *N. capensis*, which is not consistent with what was reported before by Werner et al. (2012). The number of individuals here reported is small, and the specimens were probably stressed as the duration of the measurement was short (less than 1 h) compared to other nine species analyzed. Despite this fast decrease in oxygen, it is possible to say that *N. megalops* was regulating its respiration rate. This ability may explain its persistence in the OMZ 24 h a day. Consequently, *N. megalops* must have evolved efficient adaptations to deal with low oxygen levels, such as, e.g., a high respiratory surface (gills) and/or a general low oxygen demand due to its smaller vertical migration movement. Thus, the ability of an animal to either maintain a constant oxygen uptake irrespective of the ambient oxygen levels or decrease its oxygen consumption rates when ambient oxygen levels decrease seems not to be influenced by its DVM behavior in the first place.

### Seasonal OMZ

Off Oregon (United States), in the Northern California Current System (NCCS), two euphausiid species dominate the macrozooplankton community: the oceanic *Euphausia pacifica* (Brinton, 1962) with DVM between the surface and depths of at least 250 m (Brinton, 1967) and the neritic cold upwelling-associated *Thysanoessa spinifera* (Brinton, 1962; Smith and Adams, 1988; Lavaniegos and Ambriz-Arreola, 2012). Because of its neritic lifestyle, *T. spinifera* does not migrate as deep as *E. pacifica*, but, instead, remain within the upper 100 m during day and night and swarm in summer at surface for reproduction (Brinton, 1962; Smith and Adams, 1988). This species is also known for its narrow plasticity when facing changes in the physical oceanographic conditions (Brinton, 1979). Indeed, *T. spinifera* is strongly influenced by the North Pacific Gyre Oscillation (Di Lorenzo et al., 2008; Sydeman et al., 2013). This oscillation is connected with the winds and upwelling responses (Chenillat et al., 2012) and corroborates the upwelling preference of this species. Important changes in both species’ distribution occurred during the El Niño event of 1992–1993, after which biomass of *T. spinifera* fell by more than 70% off Oregon and British-Columbia (Tanasichuk, 1999). In the southern part of the CCS (at approximately 30°N; Off Baja California), *E. pacifica* took some time to recover after the El Niño event of 1997–1998, but was abundant again during summers of 2000, 2002, and 2005. These high abundances were linked to La Niña in 2000, a sub-Arctic water intrusion in 2002 and to high upwelling conditions in 2005 (Lavaniegos and Ambriz-Arreola, 2012). It is clear that El Niño brings low upwelling conditions (low food availability) and warmer deoxygenated water, which are not optimal for the temperate species of the NCCS.

A different pattern within the respiratory response to declining *p*O_2_ was observed between *T. spinifera* and *E. pacifica*. The neritic lifestyle, short vertical migration distance, and strong association with upwelling areas (high nutrients, cold temperature, and lower oxygen concentration) match the comparatively low metabolic rate of *T. spinifera* compared to oceanic *E. pacifica*. The strong association of *T. spinifera* with upwelling conditions likely signifies an oxyconformity strategy to tolerate the typical low oxygen concentration of upwelled water. So far, no acoustic or direct observations of hypoxia and warming effects on *T. spinifera* have been reported. However, massive stranding events in several bays on the US West Coast over an area of approximately 400 km between Oregon and California were observed in summer of 2013 and related to the strongly hypoxic conditions prevailing regionally (Leising et al., 2014). This hypoxic zone was extending into the upper 50–100 m of the water column. A similar situation was observed in the Gulf of California (Mexico) with the subtropical species *N. difficilis* (López-Cortés et al., 2006), the counterpart in the Pacific of *N. megalops*. The authors proposed that high unusual upwelling conditions promoted a phytoplankton bloom, which indirectly depleted the oxygen concentration with the sinking of organic matter. This would have forced the mesopelagic *N. difficilis* to migrate upward toward more oxygenated waters and then to be washed out by the surface currents. *N. difficilis* was shown to be relatively tolerant to hypoxic conditions, but less than the tropical species *Euphausia eximia* (Tremblay et al., 2010; Seibel et al., 2016) and *Nemastocelis gracilis* (Seibel et al., 2016).

High tolerance to hypoxia was assumed in the past for *E. pacifica* because of its low critical oxygen partial pressure (*P_*crit*_* = 18 mm Hg, 2 kPa or 11% air saturation at 10°C), lower than what the species experiences *in situ* at 350 m depth in its habitat (off South California; Childress, 1975). In fjords and bays their downward migration is often reduced (Bollens et al., 1992), sometimes limited by seasonal hypoxic or anoxic conditions in bottom water layers (Kunze et al., 2006). In these environments, *P*_*crit*_ values of *E. pacifica* were higher (*P_*crit*_* = 4 kPa or 20% air saturation at 10°C), showing less hypoxia tolerance (Ikeda, 1977). The RI of *E. pacifica* indicated that this species is not an outstanding oxyregulator as BCS and polar species. The high standard deviation of RI may speak for a lack of a consistent strategy when dissolved oxygen concentration decrease at *in situ* temperature.

Alternation between El Niño and La Niña events maintains the abundance of krill across time in the NCCS, but it is clear that if strong El Niño event like the one of 1997–1998 last longer or occurs more often, both *T. spinifera* and *E. pacifica* stocks would probably disappear from the NCCS and continue their life cycle at higher latitudes in the Gulf of Alaska, where they are not so affected by the El Niño event. This would have strong consequences for the higher trophic levels of the NCCS.

### Cold Regions and OMZ-Free

The polar species *Euphausia superba* and *Thysanoessa inermis* exhibit also one of the highest RI among the 10 species assessed. The Antarctic krill *E. superba* is a central constituent of Antarctic food webs and forms large biomasses in the Southern Ocean (Atkinson et al., 2004; Murphy et al., 2007). Cumulative impacts of sea ice decline and ocean warming have negatively modified the abundance, distribution and life cycle of this species (Flores et al., 2012). The species *T. inermis* is restricted to the North Atlantic, North Pacific and the shelf region around Spitsbergen, continuously advected to the Arctic by the ocean currents from the Barents Sea where they have their major spawning ground. Both polar species are known as pronounced vertical migrators with some flexibility depending on food availability and predation risk (Kaartvedt et al., 1996; Cresswell et al., 2009).

As oxygen levels in Polar regions are relatively high, it appears that there is no compelling need to evolve adaptations to low oxygen levels. However, both species are well known for their dense swarming behavior and may experience reduced oxygen levels in these dense aggregations (Brierley and Cox, 2010). According to Brierley and Cox (2010), the oxygen concentration in a median packed *E. superba* swarm (40 m diameter, 111 ind. m^–3^) can fall from 6.8 to 5.8 mL O_2_ L^–1^ (76 to 65% air saturation or 16 to 14 kPa in South Georgia) after approximately 3 min spent in the middle. Swarm density can reach 25,000 ind m^–3^ (Hamner and Hamner, 2000) or spread over hundreds km^–2^ (Nowacek et al., 2011), so it can be easily envisaged that the reduction in oxygen availability in the middle of these biological features may be dramatically higher. This is probably the reason why *E. superba* and *T. inermis* deploy unexpected high hypoxia tolerance at *in situ* temperatures. As temperature generates higher energy demands in *T. inermis* (>three-fold), temperature rise in the North-Arctic of 3°C above the current summer conditions could lead to increased competition with other warmer adapted species, like *Meganyctiphanes norvegica* and *N. megalops* (Huenerlage and Buchholz, 2015). So far, this Arcto-boreal species seems to benefit from the current higher water temperatures in the Arctic as it seems to reproduce successfully in the Kongsfjorden (Buchholz et al., 2012).

### RI or Others?

Even though temperature increases the metabolic activity, hence the energy demands of an animal, the present study suggests that the ability to cope with low oxygen levels is not always worse at higher temperatures for hypoxia-adapted species. A possible explanation could be that despite higher energy expenditure other processes such as diffusion rates are also enhanced, providing sufficient oxygen for an animal. As a consequence, we suggest that it is of crucial importance to measure respiration rates at *in situ* temperatures when comparing the hypoxia tolerances of various species within and between ecosystems.

Furthermore, the RI value, as a proxy for the capability of an animal to withstand low oxygen levels, seems to be indicative for the oxygen tolerance for its own. Low RI values were possible to determine for species such as *E. mucronata*, *E. distinguenda*, and *E. lamelligera* as they were showing oxyconformity or metabolic suppression patterns. In contrast, *E. hanseni* and *N. capensis* occurring in the BCS show high RI values. However, all species are known to withstand comparably low oxygen levels. This highlights the need to analyze the RI, additionally to *P*_*crit*_, to get a wider understanding of the species-specific adaptation strategies. Standardization to calculate RI is important as its determination depend on the model used (Cobbs and Alexander, 2018). In the present work, in order to reduce user interpretation in calculating RI by mean of the best fitted curve, we used the area under curve of the original data sets.

The analysis of the ETS activity and the contribution of the alternative oxidase (AOX) pathway are parameters that could be implemented to understand other metabolic adaptations related to vertical oxygen and temperature gradients. High specific ETS activities were observed in zooplankton collected in the Equatorial-Subtropical Atlantic mesopelagic zone (Hernández-León et al., 2019), also coinciding with a previous observation in the Eastern Equatorial Pacific (Herrera et al., 2019). The authors discussed this observation as an adaptation of migrant zooplankton to endure the adverse conditions of low temperature and low oxygen in deep waters. The AOX pathway is also a promising avenue to explore in response to temperature and oxygen vertical gradients as it has been identified and expressed in the copepod *Tigriopus californicus* in response to cold and heat stress compared to normal rearing temperature (Tward et al., 2019). This pathway could be an important player to support partial electron transport in order to stabilize mitochondrial membrane potential during metabolic suppression of OMZ-adapted species when residing for some hours in hypoxic conditions, as seen in the gills of freshwater bivalves adapted to hypoxia (Yusseppone et al., 2018).

It is known that species or populations of species confined to one hemisphere or a particular part of the ocean (neritic vs. oceanic) become often specialists (Jones and Cheung, 2017). They are in most cases neither widely distributed nor physiologically versatile, and can be predicted to especially suffer from the effects of ocean warming and OMZs’ expansion. This may be also true for euphausiid species, but this study clearly illustrates that most euphausiids, using different strategies, cope with a range of different oxygen and temperature levels – showing high physiological plasticity – and hence, explaining why this successful taxon is predominate in all the world’s oceans. However, species from the NCCS, ETP and the Arctic may be more vulnerable to future environmental conditions with increased water temperatures and decreased oxygen levels.

## Data Availability Statement

All datasets generated for this study are included in the article/Supplementary Material.

## Author Contributions

All authors participated in the concept of the study. NT conducted the experiments, analyzed the results, draw the figures, wrote, and revised the manuscript. KH conducted the experiments and contributed to data analysis, writing and revision of the manuscript. TW conducted the experiments, analyzed the results, and contributed to writing and revision of the manuscript.

## Conflict of Interest

The authors declare that the research was conducted in the absence of any commercial or financial relationships that could be construed as a potential conflict of interest.

## Footnotes

1 www.r-project.org
